# Sporotrichosis: The Story of an Endemic Region in Peru over 28 Years (1985 to 2012)

**DOI:** 10.1371/journal.pone.0127924

**Published:** 2015-06-01

**Authors:** Max Carlos Ramírez Soto

**Affiliations:** 1 Clinical Pathology Service, Hospital Regional Guillermo Diaz de la Vega, Abancay, Peru; 2 Clinical Pathology Service, Santa Teresa Clinic, Abancay, Peru; Columbia University, UNITED STATES

## Abstract

**Background:**

Abancay province is a long-standing geographical focus of sporotrichosis in the south central highlands of Peru. Therefore, we examined the features of 36 newly identified cases of sporotrichosis from two hospital centers in Abancay province. We also performed a literature review of studies conducted in this endemic geographical focus over a period of 28 years (1998 to 2012), and analyzed the demographic, clinical and epidemiological features of sporotrichosis in the cases reported in these studies.

**Methodology:**

We examined the features of 36 new cases of sporotrichosis identified from two hospital centers in Abancay. Furthermore, we searched for relevant studies of cases of sporotrichosis in the endemic region using healthcare databases and literature sources. We analyzed a detailed subset of data on cases collected in Abancay, neighboring provinces, and other regions of Peru.

**Results:**

A total of nine studies were identified, with 1467 cases included in the final analysis. We also analyzed 36 new cases found in the two hospital centers. Therefore, the combined total of cases analyzed was 1503. Of this total, 58% were male, and approximately 62% were aged ≤14 years. As expected, most cases were from Abancay province (88%), although 12% were from neighboring provinces and other regions of Peru. The lymphocutaneous form (939 cases) was the commonest. The face was the most commonly affected region (647 cases). A total of 1224 patients (81.4%) received treatment: 95.8% received potassium iodide, 2.6% ketoconazole and 1.6% itraconazole. The overall success rates were 60.7% with potassium iodide, 32.2% with ketoconazole and 85% with itraconazole.

**Conclusions:**

The epidemic of sporotrichosis has been occurring for three decades in the province of Abancay in Peru. This mycosis affects primarily the pediatric population, with predominantly the lymphocutaneous form in the facial region. Although treatment with potassium iodide is safe and effective, response and adherence to treatment are influenced by its duration, cost, accessibility, and side effects.

## Introduction

Sporotrichosis is a subcutaneous mycosis, caused by various species of the genus *Sporothrix*, a saprophyte of organic matter, mainly in plants and vegetables [1]. Initially, the causal agent of this disease was thought to be a unique thermally dimorphic fungus *Sporothrix schenckii*. Recently, *S*. *schenckii* isolates, which exhibited a degree of geographical specificity, were regrouped into at least six cryptic species by multilocus phylogenetic analysis. Medically relevant species of *Sporothrix* now include *S*. *brasiliensis*, *S*. *schenckii* (*sensu stricto*), *S*. *globosa*, *S*. *mexicana* and *S*. *luriei* [2,3]. Infection is acquired by traumatic inoculation of environmental material, or from a cat scratch or bite [1,4–6]. After inoculation of the fungus, the individual may develop lymphocutaneous lesions, fixed or disseminated [1,7,8]. In recent decades, the incidence has been increasing in tropical and subtropical regions, with outbreaks associated with zoonotic transmission [4,5]. Sporotrichosis is now the commonest subcutaneous mycosis in the Americas [1]. In Peru, sporotrichosis occurs more frequently in the Andean provinces, including Abancay, Cajamarca, La libertad, Cusco and Ayacucho, although it is also increasingly reported in areas where the disease was rare decades ago [8–12]. Sporotrichosis is hyperendemic in Abancay, an inter-Andean province located in the Andes Mountains, where the overall incidence between 1997 and 1998 was 98 cases/100,000 inhabitants and a pediatric incidence of 156 cases/100,000 children aged ≤15 years [8,9]. The endemic area of sporotrichosis in Abancay are characterized by poor sanitation, substandard housing and little access to health services—a challenge to control and eradication of the disease. Over the last two decades, the increase in the number of cases of the disease has been continuous for more than 20 years and remains on the rise, affecting vulnerable groups. In the light of these data, we examined the features of 36 new cases of sporotrichosis from two hospital centers in Abancay province. We also performed a literature review of studies conducted in this endemic geographical focus over a period of 28 years (from 1998 to 2012), and analyzed the demographic, clinical and epidemiological features of sporotrichosis in the cases reported in these studies.

## Materials and Methods

### Study Area and Population

The study was conducted in Abancay (the departmental capital of Apurímac), a poor area in the south central highlands of Peru (72.88°W, 13.63°S, 2392 m above sea level) [13] where there are two main seasons—a warm and wet season (November to April), and a cold and dry season (May to October). From November to April, the maximum temperature (Tmax) commonly reaches 20°C (range 0–20°C) and the 6-month cumulative precipitation is 100–1000 mm; in contrast, from May to October, the Tmax rarely reaches 20°C (range -4–28°C), and the 6-month cumulative precipitation is around 0–500 mm. The Tmax usually peaks from September to October (12–32°C) [14]. The estimated population in 2013 was 105,694 according to a regularly updated census, and the major industries of Abancay are small businesses, agriculture and regional services [13].

### Report of New Cases

We performed a retrospective study of 36 newly identified cases of sporotrichosis from two hospitals in Abancay province: 26 cases from Santa Teresa Clinic (STC) in 2012 (previously, Centro Medico Santa Teresa [CMST]), and ten cases from Pueblo Joven Centenario Health Center (PJCHC) in 2011. STC is a primary care hospital, with 20-bed inpatient as well as outpatient facilities serving the city of Abancay and the provinces of Apurímac. Moreover, since 1982, STC, which is equipped with the appropriate laboratory facilities and clinical infrastructure for the diagnosis and treatment of sporotrichosis, has been the main referral center for sporotrichosis in Apurímac [7,8,15]. PJCHC is an 18-bed health center that serves both as a community hospital for the periurban area, where sporotrichosis is endemic, and as a primary care hospital in Abancay province.

The isolation of *Sporothrix* from clinical culture samples was used as an inclusion criterion. Clinical and laboratory data were retrieved from the original medical records and a form attached to the medical history. We also reviewed patients’ demographic information, diagnosis, clinical form of infection, anatomical location, evolution time of the lesions, treatment administered and adverse reactions.

### Sporotrichosis in Abancay: Literature Review

The literature review was conducted, according to the guidelines of the PRISMA statement [16], which is available in S1 Table. We performed a bibliography search of studies published in English and Spanish, without any year limit, using the following online databases: PubMed/MEDLINE, Lilacs, Embase and SCIELO. Key search terms included “Sporotrichosis”, “Abancay”, “*Sporothrix schenckii*”, “Sporotrichosis Abancay”, “Abancay children”, “risk factors for sporotrichosis” and “endemic Abancay”. Articles were also obtained through hand searches from journals relevant to infectious diseases and dermatology, conference proceedings and past reports of STC. Studies describing any form of sporotrichosis and *S*. *schenckii* infection in Abancay, and neighboring provinces were included, and studies that lacked clinical and therapeutic data, duplicate results and clinical images were excluded. We then analyzed the included studies in terms of demographic, clinical, and therapeutic features, and risk factors for sporotrichosis.

### Statistical Analysis

Continuous data are described with descriptive statistics, including mean (range) as appropriate and categorical data with frequencies (%). Results of primary studies of risk factors were described by Odds ratios (OR) and confidence intervals (CI).

### Ethics Approval

The study was approved by the institutional review boards of Institute of Tropical Diseases and Biomedicine of National University of San Antonio Abad of Cusco, Peru. Only retrospective information and anonymous data were used.

## Results

**Fig 1:**
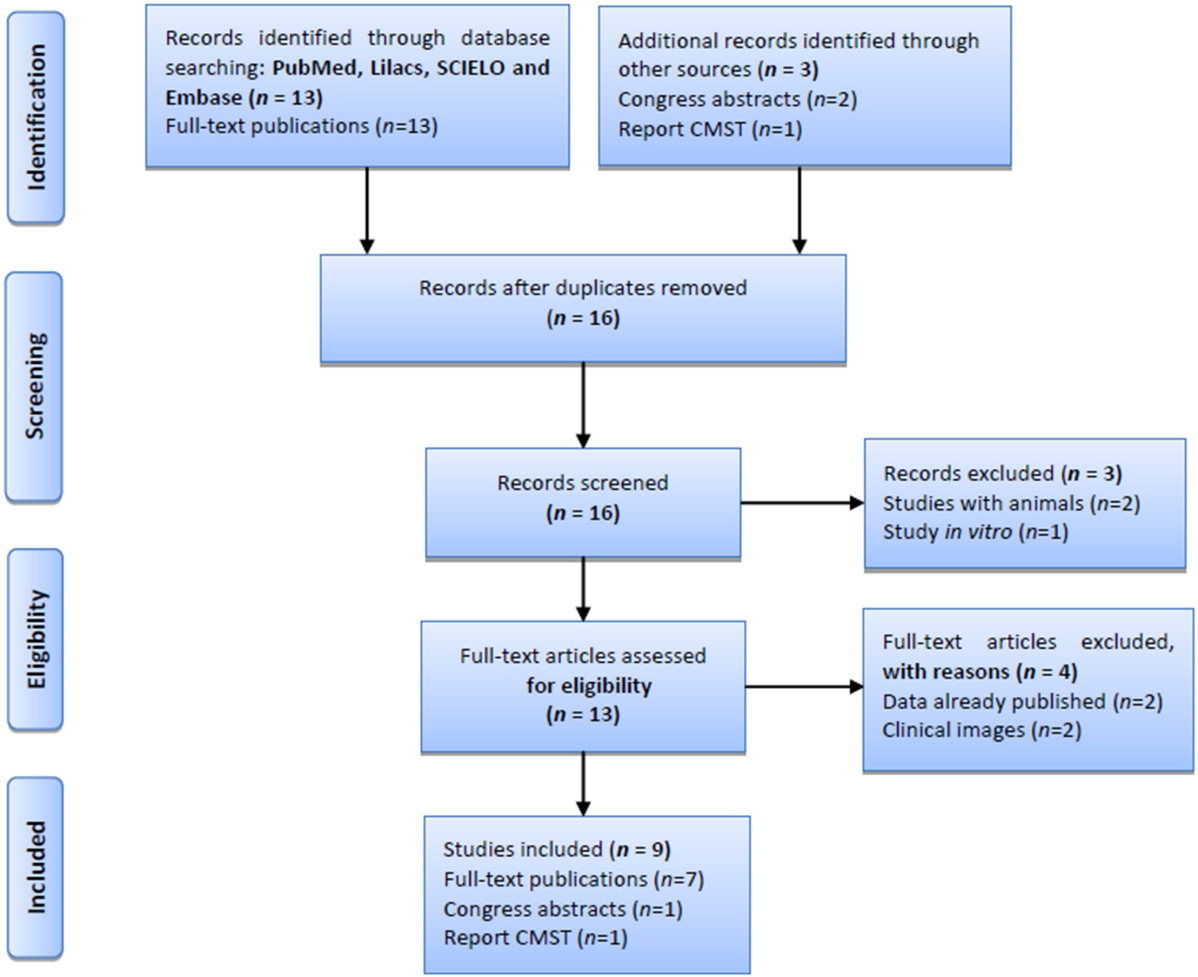
Flow diagram of literature search. CMST, Centro Medico Santa Teresa.

From the literature review, a total of 16 studies were identified, of which nine were included in the final analysis which comprised 1527 cases of sporotrichosis from 1985 to 2011 [7–9,15,17–21] (Fig 1). Of the 1527 cases, data from only 1467 cases were used, as they included complete demographic and clinical information. Therefore, together with the 36 new cases from the two hospital centers, we analyzed a total of 1503 cases (Table 1).

**Table 1:** Cases of sporotrichosis in Abancay and main characteristics of the studies analyzed.

First Author [Ref] (Years)	No. of patients/ Mean age (range, yr)	Place of study	Period of data collection	Age (yr)		Gender		Clinical form, n (%)		
				0–14	≥15	M	F	Lymphocutaneous	Fixed	Disseminated
Present study	26/ 25 (2 to 82 yr)	STC	2012	14	12	14	12	13 (50%)	13 (50%)	0
Present study	10/ 20 (3 to 65 yr)	PJCHC	2011	6	4	4	6	6 (60%)	4 (40%)	0
Ramírez et al. [7] (2012)	485/ 18 (6 m to 86 yr)	STC	2004–2011	276	209	302	183	308 (63.5%)	176 (36.3%)	1 (0.2%)
Aguilera et al. [17] (2005)	198/ (0–14 and ≥15 yr)	CMST	2000–2003	138^a^	60	68	70	83 (60%)	41 (30%)	14 (10%)
Lyon et al. [9] (2003)	201/ 10 (1 to 82 yr)	3 Laboratory Abancay	1997–1999	133	68	127	74	201 (100%)^b^		
Pappas et al. [8] (2000)	238/ (0–14 and ≥15 yr)	CMST	1995–1997	143	95	133	105	130 (55%)	85 (36%)	23 (9%)
Meneses et al. [18] (1992)	18/ (9 to 57 yr)	CMST	1990	not specif		10	8	10 (55.6%)	7 (38.9%)	1 (5.5%)
Cabezas et al. [15] (1996)	57/ (2–16 and 3–18 yr)	CMST	1990	2–16 yr (*n* = 29) 3–18 yr (*n* = 28)		30	27	26 (45.6%)	30 (52.6%)	1 (1.8%)
Meneses et al. [19] (1991)	261/ (0–24 and ≥25 yr)	CMST	1987–1989	0–24 yr (*n* = 205) ≥25 yr (*n* = 56)		153	108	138 (52.9%)	97 (37.1%)	26 (10%)
Flóres et al. [20] (1991)	39/ (0 to 49 yr)	CMST	1986	26	13	15	24	13 (33.4%)	22 (56.4%)	4 (10.2%)
Reports STMC [21] (1985)	30/ (0–14 and ≥15 yr)	CMST	1985	19	11	15	15	11 (36.7%)	15 (50%)	4 (13.3%)

Abbreviations: yr, years; m, months; M, male; F, female; STC, Santa Teresa Clinic; CMST, Centro Medico Santa Teresa; PJCHC, Pueblo Joven Centenario Health Center.

^a^The study included only children aged 0–14 years.

^b^The study included only cases of lymphocutaneous sporotrichosis.

**Fig 2:**
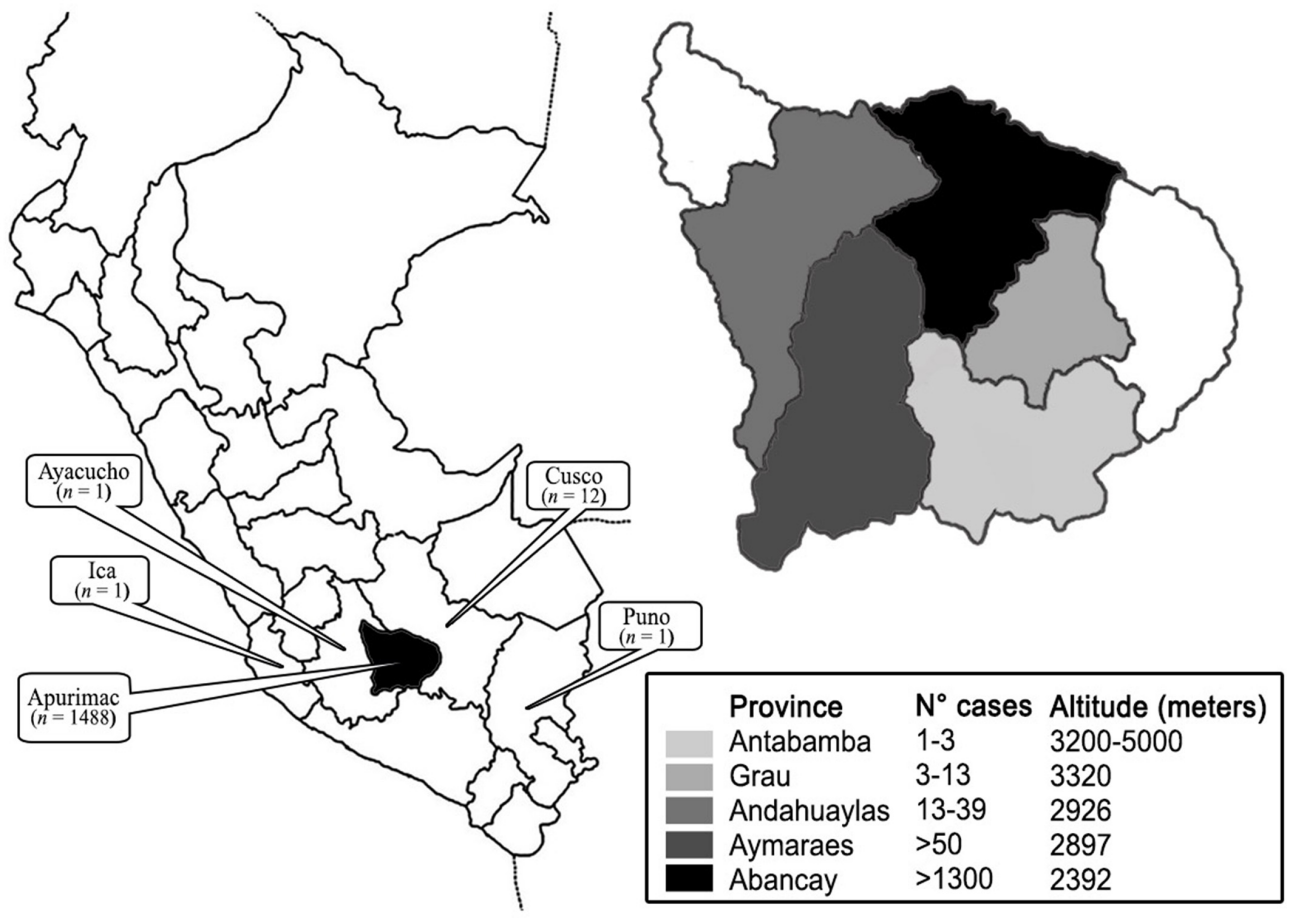
Geographical distribution of sporotrichosis cases in Abancay—Apurímac 1985–2012, Peru. The findings reported a lower number of casos at higher altitude in Apurímac.

Of the 1503 cases, 58% (n = 871) were men, and 42% (n = 632) women, aged between 6 months and 86 years, with approximately 62% aged ≤14 years. In addition, 88% (n = 1318) of the cases were from Abancay province, while patients from the neighboring provinces (Andahuaylas, Aymaraes, Antabambas and Grau) accounted for 11% (n = 175) of the cases, and 1% (n = 15) were from other regions of Peru (Cusco, Ayacucho, Ica and Puno) (Fig 2). Over 70% were preschool children and school students, 5.4% farmers and 3.7% housewives. Other occupations included administrative roles, builders, craftsmen, taxicab drivers and peddlers.

The clinical lymphocutaneous form (939 cases, 62.5%) was the commonest form, followed by the fixed (490 cases, 32.6%) and disseminated (74 cases, 4.9%) forms (Table 1). The evolution time of the lesion to diagnosis ranged from 7 days to 5 years. The location of the lesions was described in 1475 (98%) patients. The lesions were reported to occur more frequently on the face (647 cases, 44%), followed by the upper limbs (524 cases, 36%), lower limbs (197 cases, 13%) and other anatomical regions (107 cases, 7%).

The diagnosis was based on the isolation of the fungus from cultures of biological samples. In the nine studies reviewed, all isolates were identified as *S*. *schenckii*. Of the 36 new cases, 34 isolates were identified as *Sporothrix* spp., and two as *S*. *schenckii* s. str. [22], using the morphophysiological method of Marimon et al. [2,3].

Furthermore, a total of 1224 patients (81.4%) received some form of medical treatment; 1173 (95.8%) received potassium iodide (KI), 31 (2.6%) ketoconazole and 20 (1.6%) itraconazole. The overall treatment success rates reported were 60.7% (n = 712) with KI, 85% (n = 17) with itraconazole and 32.2% (n = 10) with ketoconazole. Details and results of these treatments are summarized in Table 2. The average treatment duration was 41.9 days with KI, and 54.7 days with itraconazole. Treatment dropout rates were 31% (n = 394) with KI, 61.3% (n = 19) with ketoconazole and 10% (n = 2) with itraconazole. Of the 1173 patients who were treated with KI, 79 (6.7%) had mild adverse events, whereas, of the 20 patients who received itraconazole, ten (50%) had mild adverse events. Treatment was discontinued due to serious adverse effects in 52 (4.4%) patients taking KI, and in two patients on ketoconazole. Itraconazole was discontinued in one patient who became pregnant, while, in another patient, the treatment with KI failed (Table 2).

**Table 2:** Treatment of cutaneous sporotrichosis in Abancay, Peru.

First Author [Ref] (Years)	No. of patients	Place of study	Treatment received [n =]	Cure rate, n (%)	Dropout rate, n (%)	Side-effects^a^	Suspended treatment
Present study	26	STC	KI 20 or 40 drops/tid [n = 22]	10 (45%)	12 (55%)	6 cases	
Present study	10	PJCHC	KI 20 or 40 drops/tid [n = 10]	6 (60%)	4 (40%)		
Ramírez et al. [7] (2012)	485	STC	KI 20 or 40 drops/tid [n = 483];	400 (82.8%) KI;	66 (13.7%) KI		17 KI^b^
			Itra 100 mg po/bid [n = 2]	2 (0.41%) Itra			
Aguilera et al. [17] (2005)	138^c^	CMST	KI 20 drops/tid [n = 138]	59 (42%)	79 (58%)	not specif	
Lyon et al. [9] (2003)	201	3 Laboratory	KI 20 or 40 drops/tid [n = 34]	not specif	not specif	not specif	not specif
		Abancay					
Pappas et al. [8] (2000)	238	CMST	KI 20 or 40 drops/tid [n = 165];	44 (26.7%) KI;	89 (54%) KI;	32 KI	(32 KI; 2 Ket)^b^
			Ket 400–800 mg/day po [n = 31]	10 (32.5%) Ket	19 (66%) Ket		1 failed KI
Meneses et al. [18] (1992)	18	CMST	Itra 100 mg/day [n = 14];	15 (83%)	2 (11.1%)	9 cases	1 pregnancy
			Itra 150 mg/day [n = 3];				
			Itra 200 mg/day [n = 1]				
Cabezas et al. [15] (1996)	57	CMST	KI 3.88 g/day [n = 29] vs.	26 (89.7%) KI [3.88 g/day]	2 (10.5%)	28 cases	3 KI^b^
			KI 20 or 40 drops/tid [n = 28]	vs. 25 (89.2%) KI			1 case lost
				[20 or 40 drops/tid]			
Meneses et al. [19] (1991)	261	CMST	KI 20 or 40 drops/tid [n = 195]	117 (60%)	66 (33.8%)	not specif	12 cases lost
Flóres et al. [20] (1991)	39	CMST	KI 20 or 40 drops/tid [n = 39]	15 (38.5%)	24 (61.5%)	4 cases	
Reports STMC [21] (1985)	30	CMST	KI 20 or 40 drops/tid [n = 30]	10 (66.6%)	20 (33.4%)	not specif	

Abbreviations: STC, Santa Teresa Clinic; CMST, Centro Medico Santa Teresa; PJCHC, Pueblo Joven Centenario Health Center; KI, Potassium iodide (Paediatric dose: 2–20 drops/tid; Adult dose: 4–40 drops/tid); Itra, itraconazole; Ket, ketoconazole; tid, 3 times daily; bid, twice per day; po, orally.

^a^Mild adverse event: metallic taste and gastrointestinal intolerance (Nausea, vomiting and gastritis).

^b^Serious adverse event.

^c^The study included only children aged 0–14 years.

**Fig 3:**
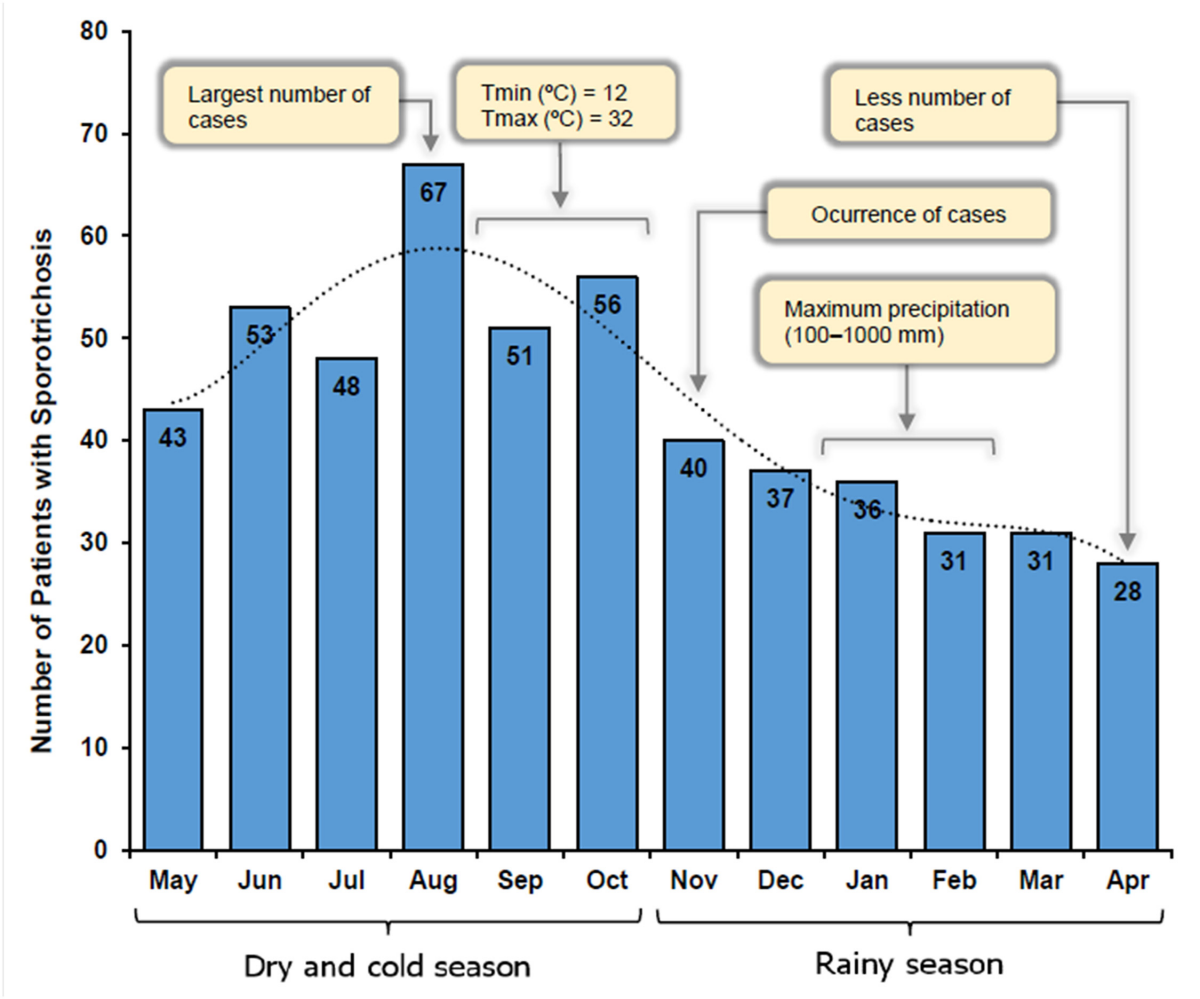
Monthly distribution of 521 cases of sporotrichosis in Abancay, Peru, from January 2004-December 2012. A strong seasonality in the cases distribution was observed between May and October (Relationship between maximal temperatures and largest number of casos). By contrast, the lowest number of cases coincides with the months from November to April (Rainy season reduces the number of cases sporotrichosis in Abancay). Tmin: Minimum temperature; Tmax: Maximum temperature.

The highest frequency of cases was noted from May to October (61%), and the lowest in April (5.4%) (Fig 3). Risk factors for sporotrichosis identified in Abancay are detailed in Table 3.

**Table 3:** Risk factors for sporotrichosis in Abancay, Peru.

Risk factor(s)	Ref.	*p* value	OR (95% CI)
Adults ≥15 years			
Ceiling made of raw wood	[9]	0.02	6.9 (1.4–35.2)
Work outdoors	[9]	0.004	5.2 (1.7–15.8)
Children <15 years			
Play in the fields	[9]	0.04	25.2 (1.1–588)
Having a cat	[7,9]	0.02	9.1 (1.3–61.4)
Dirt floors in the house	[7,9]	0.04	3.8 (1.0–13.7)
Ownership of a pig	[9]	0.006	0.03 (0.002–0.3)
Ownership of a radio	[9]	0.02	0.02 (0.001–0.5)

## Discussion

The first 15 cases of sporotrichosis in Abancay were found in Santa Teresa Clinic (previously, Centro Medico Santa Teresa [CMST]) between 1982 and 1984. These findings led us to establish a program of diagnosis and treatment in this province [21], and STC has since been treating a steadily growing number of patients with sporotrichosis. In late 2012, more than 1500 patients were diagnosed with sporotrichosis [7–9,15,17–21], with the most frequently affected group being males aged ≤14 years (62%), which explains the high incidence in the preschool and school population (>70%). This is in contrast to Rio de Janeiro where women aged >30 years are the most affected [4,6].

In our study, we found the largest number of cases was from Abancay (88%). In this province, the highest frequency of cases coincided with the dry and cold season (May to October; 61%), and the lowest frequency in the rainy season (November to April; 39%) (Fig 3). Contrary to this, other studies in Himachal Pradesh and South Africa reported rain and humidity as two significant favourable factors for sporotrichosis [23,24]. There is very limited evidence that sporotrichosis is seasonal [1]. However, our study findings suggest that climatic factors might play a role in the distribution and frequency of sporotrichosis, as, if the climate in Abancay were to become hotter and drier (due to either natural variability or anthropogenic forcing), this could potentially influence the distribution and severity of sporotrichosis. We identified reports of cases in neighboring provinces, suggesting that the increase in infection is not limited to Abancay (Fig 2). In this Andean region, Apurímac has inhabitants spread across all ecological zones, and the distribution of sporotrichosis may be influenced by the heterogeneity of climatic and geographical conditions and the high number of farmers in these provinces. Therefore, the geo-climatic conditions are conducive to the growth of *Sporothrix* as a saprophyte in the environment. Sporotrichosis is also prevalent in other Peruvian agricultural and rural areas [10–12], as was observed in 1% of cases.

As reported in our clinical findings here and from other studies, the lymphocutaneous form is the commonest clinical form (62.5%) [1,4–9]. In contrast, the fixed form is less common and morphologically has a wide clinical spectrum, ranging from lesions with ulcerated plaques, crusted, warty, and satellite lesions to psoriasiform lesions [1,22]. This is associated probably with the virulence of the strain, the immune status and depth of inoculation, and the occurrence of multiple inoculations in patients from endemic areas during their work or recreational activities. The disseminated form occurs in patients with diabetes, HIV/AIDS, hematologic malignancies, chronic alcoholism and malnutrition [1,25–27]. It is possible that the smaller number of cases in our series (4.9%) showing disseminated lesions included individuals from these patient groups.

In our study, about half of the cases had facial involvement (44%), mainly children aged ≤14 years. This finding could be attributed to the fact that the face is an exposed area with delicate skin, susceptible to superficial trauma or scratching with fingernails laden with contaminated soil [7,8]. Lesions on the upper limbs were present in 36% of patients; again these are areas that are exposed, and therefore susceptible, to inoculant trauma, especially in people aged ≥15 years, such as farmers, who manipulate plant materials [7,9]. On the other hand, the lower limbs and other anatomical regions were less affected.

**Fig 4:**
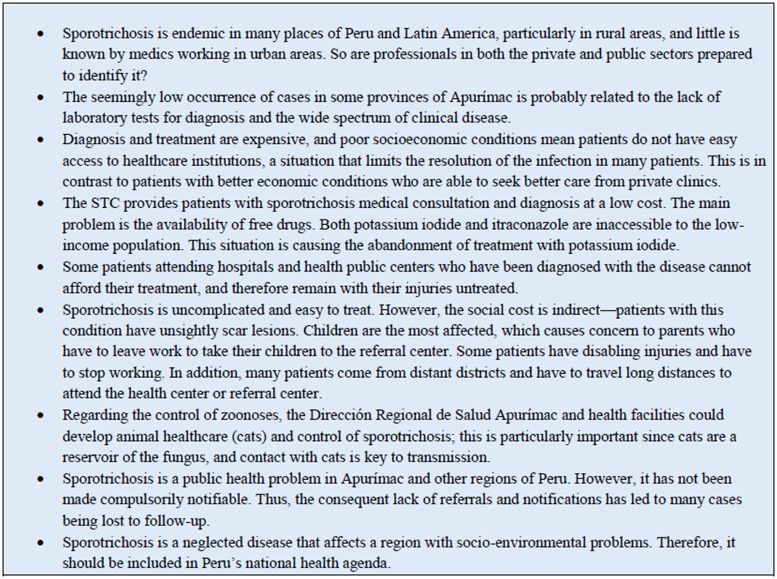
Health and current situation of sporotrichosis.

The drug of choice for sporotrichosis is itraconazole [28]. However, in Abancay, KI has been adopted as the treatment of choice, starting with two drops, and a maximum of 20 drops/3 times daily in children and 4–40 drops/3 times daily in adults [7,15], with the duration of treatment determined by the evolution of the lesions [7,15,19,20]. In the current study, the therapeutic response to KI was moderate; of the 1173 patients who received KI treatment, 60.7% were cured, and adverse effects were also lower. Of note, there is a lack of clinical trials reported in the literature to determine the optimal dose of KI as a treatment for sporotrichosis. The results of treatment with itraconazole and ketoconazole were not convincing, because these were testing treatments. Dropout rates to treatment were high, probably due to the treatment duration, cost, accessibility, and side effects (Fig 4)

Two isolates from our 36 new cases were identified as *S*. *schenckii s*. *str*. [22], whereas all isolates from the nine studies were identified as *S*. *schenckii*. In 2004, Holechek et al. identified six genotypes of *S*. *schenckii* in clinical samples from patients of Abancay, which he named genotypes I, II, III, IV, V and VI [29]. These results suggest that, in Abancay, there could exist a high variability of the *Sporothrix* species, consistent with the species of the *Sporothrix* complex described by Marimon et al. [2,3].

It is still not certain how the infectious agent has been disseminated throughout Abancay and its outskirts. In this province, patients with poor socioeconomic status live in mud houses with dirty floors, thatched or wooden, and in close proximity to thorny plants, all of which provide a favorable environment for fungal growth. In adults, the risk of infection increases when working in the field without protection (men and women cultivate and plough the land, and collect firewood and fodder from forests, as well as rear livestock) and with children playing in the field [7–9]. Thus, people are at high risk of acquiring the disease. Such practices are likely to promote the continued spread of the mycosis, a problem which must be controlled in Abancay. Interaction with cats is another key risk factor to the transmission of the fungus, which can lead to epidemic outbreaks [4–6]. In 2008, Kovarik et al. [30] isolated the fungus on claw fragments and materials collected from cats’ nasal and oral cavities. These findings suggest that apparently healthy cats are a reservoir of the fungus and can transmit the infection without showing outward signs of the disease [30]. Indeed, contact with cats has been shown to be a significant risk factor for sporotrichosis in this endemic geographical focus of Peru [9]. Moreover, given the high proportion of cases, the wide geographical distribution in the regional territory, and the high frequency of contact with cats, it is possible that an epidemic outbreak could result from this zoonotic transmission, similar to the situation in Brazil which began in 1998 and today is still uncontrolled [4–6]. Finally, the current status of sporotrichosis is also influenced by the prevailing health situation (Fig 4).

## Conclusions

The epidemic of sporotrichosis has been occurring for three decades in the province of Abancay in Peru. This mycosis affects primarily the pediatric population, predominantly as the lymphocutaneous form in the facial region. Although treatment with KI is safe and effective, response and adherence to treatment are influenced by its duration, cost, accessibility, and side effects. Therefore, this disease should be included in Peru’s national health agenda.

## Supporting Information

S1 Table PRISMA 2009 Checklist. (DOC)
